# Supplementary material for: Exploring chromosomal structural heterogeneity across multiple cell lines
Source: eLife. 2020 Oct 13;9:e60312. doi: 10.7554/eLife.60312 (PMC7593087; doi:10.7554/eLife.60312)
Supplement: Supplementary file 1. — The energetic parameters are provided in units of ε. We consider five chromatin types (A1, A2, B1, B2, and B3) and a non-specific type (NA). [file elife-60312-supp1.docx]

**MiChroM parameters for type-to-type interactions in units of ε.**

|  | A1 | A2 | B1 | B2 | B3 | NA |
| --- | --- | --- | --- | --- | --- | --- |
| A1 | -0.268028 | -0.274604 | -0.262513 | -0.258880 | -0.266760 | -0.225646 |
| A2 | -0.274604 | -0.299261 | -0.286952 | -0.281154 | -0.301320 | -0.245080 |
| B1 | -0.262513 | -0.286952 | -0.342020 | -0.321726 | -0.336630 | -0.209919 |
| B2 | -0.258880 | -0.281154 | -0.321726 | -0.330443 | -0.329350 | -0.282536 |
| B3 | -0.266760 | -0.301320 | -0.336630 | -0.329350 | -0.341230 | -0.349490 |
| NA | -0.225646 | -0.245080 | -0.209919 | -0.282536 | -0.349490 | -0.255994 |
